# Environmental relevant concentrations of copper sulphate induce biochemical and molecular toxicity in *Labeo rohita*

**DOI:** 10.1371/journal.pone.0328238

**Published:** 2025-07-28

**Authors:** Saima Naz, Riaz Hussain, Hafiz Muhammad Ali, Nasir Masood, Ghazala Jabeen, Rehana Iqbal, Momil Liaquat, Muhammad Irfan Ullah, Kashif Hussain, Tuğçe Merve Berberoğlu, Ahmed A. El-Mansi, Eman A. Elbealy, Abdelalim A. Gadallah, Kasim Sakran Abass

**Affiliations:** 1 Department of Zoology, Government Sadiq College Women University, Bahawalpur, Pakistan; 2 Faculty of Veterinary and Animal Sciences, The Islamia University of Bahawalpur, Bahawalpur, Pakistan; 3 Department of Biosciences, COMSATS University Islamabad, Islamabad, Pakistan; 4 Department of Zoology, Lahore College for Women University, Lahore, Pakistan; 5 Institute of Zoology, Bahauddin Zakariya University, Multan, Pakistan; 6 Department of Pathobiology, Faculty of Veterinary Sciences, Bahauddin Zakariya University, Multan, Pakistan; 7 Department of Pathobiology and Biomedical Sciences, Faculty of Veterinary and Animal Sciences, MNS University of Agriculture, Multan, Pakistan; 8 Department of Animal Nutrition and Nutritional Diseases, Faculty of Veterinary Medicine, Kafkas University, Kars, Türkiy; 9 Biology Department, Faculty of Science, King Khalid University, Abha, Saudi Arabia; 10 Biology Department, College of Science, Jazan University, Jazan, Saudi Arabia; 11 Department of Physiology, Biochemistry and Pharmacology, College of Veterinary Medicine, University of Kirkuk, Kirkuk, Iraq; Benha University, EGYPT

## Abstract

Copper is an important element involved in the catalysis of many vital reactions in the body of an organism. However, excessive copper causes cellular damage by accelerating the production of reactive oxygen species and disrupting the physiological reactions. The present research was conducted to determine the toxicological effects including oxidative stress profile, concentrations of anti-oxidant enzymes and genotoxicity of three different doses (0.28 µg/L, 0.42 µg/L and 0.56 µg/L) of copper sulphate (CuSO_4_) subjected to *Labeo rohita* for 36days. Micronucleus test indicated a significant (p < 0.05) increase in the frequency of morphological and nuclear changes in the erythrocytes of the treated fish. A significant (p < 0.05) increase was observed in oxidative stress parameters (ROS, TBARS) whereas the activity of anti-oxidant enzymes (SOD, POD, GSH, CAT) was significantly (p < 0.05) decreased in the gills, brain, liver and kidneys of fish exposed to 0.56 µg/L concentration of CuSO_4_. Moreover, CuSO_4_ exhibited significant (p < 0.05) DNA damage in lymphocytes, brain cells, hepatocytes and renal cells, as determined by comet test. Hence, it has been concluded that CuSO_4_causesevere biochemical and physiological disruptions in different organs of *Labeo rohita*, hence, considered as hazardous even at very low sub-lethal concentrations.

## Introduction

Industrial sector is the backbone of the development of a country; however, its untreated effluents are damaging for the environment, since at every day, huge amount of municipal, industrial and agricultural wastes are being released in the water environment causing serious ecological problems [1,2]. These released heavy metals accumulate in liver, gills, kidneys, intestine, integument and muscular tissue inthe body of aquatic animals [3,4], thus posing serious concerns not only for their health and well-being but also for the consumers [5–8]. The body exposure to metals like cadmium (Cd), mercury (Hg), lead (Pb), copper (Cu), nickel (Ni), zinc (Zn) and arsenic (As) over an extended period of time affects the organisms by disrupting their cellular functions like growth, proliferation, differentiation, tissue repair processes and thus, cause apoptosis, hepato-renal, skin and cardio-toxicities [9,10]. It has been found that the concentration of Cu (2.14–2.17 mg/L) is relatively higher among the metals, i.e., As (0.015–0.40 mg/L), Cd (0.02–0.029 mg/L), cobalt (Co; 0.31–0.38 mg/L), Cr (1.02–1.09 mg/L) and Hg (0.01–0.04 mg/L) in the groundwater and these levels are considered to be higher than the acceptable threshold values given by WHO [11]. Except the metals like Zn that are essentially required for biological and chemical processes of the body but only in minute concentrations, the bioaccumulation of As, Cd, Cu, Pb, thallium and Zn was observed in gills, liver, kidneys, skin and muscles of different fish like *C. aumtus*, *X. Argentea*, *P. fulvidraco* and *O. mossambicus* [3,12–14] that impart detrimental effects on physiological performance of birds [15] and animals [16,17].

Copper is an important metal, present at plentiful concentrations in mines and the environment (5000ng/m^3^) [18,19], abundant in rivers [20,21], coastal systems [22] and estuaries [23]. It plays a significant role in critical enzyme reactions in the physiological processes of both human and animals as a co-factor [24,25], is an important biological unit in the oxygen transportation as part of hemocyanin and also a counterpart of hemoglobin (Hb) for oxygen transportation found in shellfish and mussels [26]. Moreover, Cuis an essential element for Hb synthesis, required for blood coagulation, bone formation, proper mitochondrial function, cellular signalling pathways like detoxification of reactive oxygen species and metabolic reprogramming for differentiation of hematopoietic progenitor cells into diverse cell lineages [19,27] and also an important micro-nutrient needed for cellular functions of vertebrates, invertebrates and aquatic species [25]. However, prolonged exposure to Cu has a number of detrimental biological and physiological effects on organisms, especially fish, including alterations in the blood chemistry, organ histo-pathology and the formation of metallothionein [25]. Cu reaches the aquatic systems through anthropogenic sources including water contamination via industrial wastes, discharge of coolant and municipal/sewage water, manufacturing discharge from pulp and paper board mills, foundries, anti-fouling paints industries and petroleum refining, mine drainage, combustion of coal (fly ash) and oil, corrosion of pipelines, use of copper salts in controlling the aquatic vegetation, application of copper fungicides and influx of Cu containing fertilizers for different crops [28,29]. Copper and other heavy metals accumulate in the body of aquatic organisms and eventually are transmitted to the humans via consumption of affected fish and other sea foods, resulting in severe gastrointestinal, renal and immune system dysfunctions, acute or chronic sickness, nervous system disorders, skin and vascular damages, birth defects and different cancers [30,31].

Fish is a critical bio-indicator for assessing the contamination of heavy metals in the aquatic habitat, since, it is in direct contact with water,have good sensitivity and potential to bio-accumulate the environmental pollutants present in water, even at quite low concentrations and thus, reflects the overall exposure to water pollutants by alterations in its physiological parameters [32,33]. Numerous physiological changes occur in the fish like production of ROS when the natural circumstances of the aquatic habitat alter due to contamination with heavy metals [28,34]. Fish have an anti-oxidative defense system that utilizes both enzymatic and non-enzymatic mechanisms to reduce the detrimental effects of ROS [6]. The decreased anti-oxidant activity along with increased oxidative stress parameters are important indicators of contamination of the aquatic life [35–37] and these stress indicators are commonly used in eco-toxicology as an early warning to assess the potential detrimental changes in the exposed animals [38–41]. Moreover, different biological markers like level of circulating exosomes [42], hepatic enzymes [43,44], anti-oxidants [45–48], hemato-biochemical parameters [49–51] and histo-pathological changes in the visceral organs [52–55] have previously been described, to establish a relationship between the effects of exposure of hazardous chemical with their toxicological effects in fish and other animal species. Additionally, current trends in molecular biology and their applications to develop a number of sensitive and selective DNA-based eco-genotoxicity tests like micronucleus test and comet assay are considered as effective, reliable and extensively used methods to identify the genotoxic effects [19,41,56,57].

The concentration of Cu has been reported to be 0.5 µg-1 mg/L (median value: 0.01 mg/L) in surface water in various studies in USA, 6 µg/L (3–19 µg/L) in the River Stour in United Kingdom and 0.8–10 µg/L in an unpolluted zone of River Periyar in India [58] or was found to be quite low (usually <20µg/L) in the surface waters [59]. Moreover, it has been reported that naturally Cu has been found to be 0.03-0.23g/L and 0.20-30g/L in seawater and freshwater systems, respectively [60]. Cu is readily accumulated in the body of different marine plants and animal species including fish, with a bio-accumulation factor ranging from 100 to 26000 and thus, leads to severe toxicological effects when this uptake exceeds the safe limits of physiological or biochemical detoxification and rate of excretion [61]. In this context, it was observed that marine amphipod *Allorchestes compressa* accumulated 100 mg/kg of copper and lead to reduced growth when exposed to only 10 µg/L of Cu for 28 days [62]. Hence, for the same reason, different low concentrations of CuSO_4_ were selected in the current project, to determine the effects of these water environment relevant concentrations of Cu on different physiological parameters of the fish, only for a short period of time, i.e., about 01 month. The experimental doses were selected to be of sub-lethal concentrations and were quite below than LC_50_ of CuSO_4_ for *L. rohita*: 3.15 mg/L [63] and 1.60 mg/L [64]. As a part of the current study, our research group has recently reported severe health effects of CuSO_4_ on hemato-biochemical profile of *L. rohita* in connection with histo-pathological alterations in different organs at these low concentrations [65]. However, the literature is deprived-off the information about the oxidative stress caused by alterations in these organ tissues of *L rohita* due to CuSO_4_ toxicity. Thus, the present study was conducted to assess the oxidative stress profile (ROS, TBARS) and concentrations of different anti-oxidant enzymes (GSH, SOD, CAT, POD) in liver, brain cells, gills and kidneys to determine the toxicological and genotoxic effects of copper sulphate in *L. rohita*.The current toxicological observations will enhance our understanding about the underlying molecular mechanisms causing breaks in the DNA strands and genome instability leading to different patho-physiological effects and in consistent molecular mechanisms that eventually adversely affects the growth and development of the organ systems and hence, ultimately severely diminish the production potential of the fish.

## Materials and methods

The experiment was conducted at the Department of Zoology, The Government Sadiq College Women University, Bahawalpur, as described earlier [65]. This study and the animal experimentations were approved by the Institutional Review Board, The Government Sadiq College Women University, Bahawalpur, Pakistan (124/20/GSCWU).

### Experimental fish and treatments

A total number of 72 freshwater fish (*Labeo rohita*) with similar body weight (200–215 g) were obtained from the Punjab Fisheries Department, Bahawalpur. After collection, the fish were placed in 100L tap water glass aquaria at standard housing conditions under controlled temperature (24 ± 1ºC), pH (7.5)and under continuous aeration at dissolved oxygen level of 6.16 ppm using aquarium air pump (SOBO SB-548A), as previously described [65]. Physicochemical characteristics of water were monitored before the start and during the whole duration of the experiment, by recording the parameters at each stage to provide a continuously comfortable environment to the fish (Table 1).

**Table 1:** Physical and chemical characteristics of water during the experimental period.

Water quality Parameters	Time of experimental period		
	Start	Mid	End
pH	7.40 ± 0.02	7.30 ± 0.03	7.50 ± 0.02
Calcium (mg/L)	39.10 ± 0.31	38.90 ± 0.83	39.20 ± 0.67
TH (CaCO_3_,mg/l)	168.90 ± 2.19	169.70 ± 1.73	171.50 ± 1.33
EC at 25^o^C (μmhos/cm)	398.70 ± 4.11	401.20 ± 2.55	403.10 ± 2.19
DO (mg/L)	7.910 ± 0.09	7.83 ± 0.13	7.89 ± 0.07
Water temperature (°C)	25.70 ± 0.15	25.60 ± 0.23	25.70 ± 0.39
TDS (mg/L)	181.50 ± 2.31	179.90 ± 2.77	181.30 ± 2.91
Chlorides (mg/L)	9.39 ± 0.05	9.49 ± 0.05	9.45 ± 0.07
Sodium (mg/L)	10.90 ± 0.11	10.81 ± 0.25	10.77 ± 0.33
Alkalinity (mg/L)	175.90 ± 0.51	177.10 ± 0.37	178.30 ± 0.27
Potassium (mg/L)	1.40 ± 0.13	1.40 ± 0.19	1.50 ± 0.09

TH = Total hardness, EC = electrical conductivity, DO = dissolved oxygen, TDS = total dissolved solids.

The fish were offered commercially available standard feed twice daily containing 25% crude protein in the form of pellets (3% of body weight) twice every day. For acclimatization, the fish (n = 72) were kept untreated for 15 days and then randomly assigned in to four treatment groups (n = 18/ group); group A was kept as control and the groups B to D were treated with CuSO_4_solution:B = 0.28 µg/L, C = 0.42 µg/L and D = 0.56 µg/L dissolved in fresh clean drinking water. The water of the aquaria was replaced on regular basis to remove the residual feed and the fecal material from the aquaria, to keep the environment clean and healthy, with a fresh addition of CuSO_4_ to maintain its constant level during the whole duration of the experimental period (36 days). All of the chemicals used in this experiment were of analytical grade and were purchased from Sigma Aldrich (USA).

### Collection of samples and biochemical assays

The treated fish (n = 6/ group) of each experimental group were selected for collection of blood and different organ tissues at days-12, 24 and 36 of the experiment. The animal suffering has been reduced by using clove oil (4.5 mg/L), to anaesthetize the experimental fish prior to blood and tissue sample collection [66]. Then, to further reduce the pain and distress to the fish, the blood (2.5mL/ fish) was drawn from caudal vein of each fish by using 26 gauge disinfected hypodermic needle. The collected blood (0.5mL) was then placed in EDTA-coated glass vacutainers, until further hematological analysis. The remaining blood (2.0mL) was kept in anti-coagulant free tubes, was centrifuged at 5000 rpm for 5 min, the serum was separated and preserved at −20ºC until further biochemical analysis [67,68].

After collection of blood, the treated fish were dissected and 0.5–1.0 cm tissue samples of heart were collected for histo-pathological examinations and liver, brain, gills and kidneys were collected for genotoxic studies [69–71]. The tissue pieces of heart were preserved in 10% neutral buffered formalin solution until further processing [72–75]. These tissue samples were processed by paraffin sectioning technique and 4–5μm thick histological sections were stained by Hematoxylin and Eosin staining technique [76–78]. All the histo-pathological observations are an average of three independent slides for each tissue.

### Genotoxicity of erythrocytes

At the time of sampling, a fine thin blood smear was prepared on a microscope slide by a drop of freshly collected blood of each treated fish, to analyze the morphological and nuclear alterations in the erythrocytes of the treated fish [79]. The smear was quickly dried, fixed with absolute methyl alcohol, stained with Giemsa stain and a total of 1000 red blood cells (RBCs) per smear per fish in each treatment group were observed under oil immersion lens (1000X) of light microscope and percentile rate was computed to determine the morphological and nuclear changes in the erythrocytes [8,80–82].

### Comet assay for genotoxicity

To assess the DNA damage, the blood lymphocytes, hepatocytes, cells from brain, gills and kidneys were separated, according to previously published method [79]. The frequency of DNA damage in these cells was measured using single cell gel electrophoresis/comet assay under alkaline conditions, as described earlier [8,83].

### Determination of oxidative stress parameters and anti-oxidant enzymes

For assessment of oxidative stress parameters and concentration of anti-oxidant enzymes, the tissue specimens were collected at days-12, 24 and 36 of the trial. Briefly, the tissue homogenates were prepared, separately from each organ tissue (brain, liver, kidneys and gills) using cold saline solution and different parameters of oxidative stress and anti-oxidant enzymes were estimated by measuring the optical density at specific relevant wavelengths in spectrophotometer, as described earlier [54].

### Reactive oxygen species (ROS)

ROS were measured by following the procedure described earlier [84]. Briefly, two different reagents were prepared; the reagent 1,Diethyl para phenylenediamine sulfate (1 mg) was mixed in 10mL deionized water, while reagent 2 was prepared by mixing 50 μL ferrous sulfate stock solution (100 mg FeSO_4_ in 20mL of sodium acetate buffer; pH 4.8) in 100mL of sodium acetate buffer. Then, both reagents were mixed at a ratio of 1:25 and placed in the dark for 2 min. Finally, about 70 μL tissue homogenate was mixed in 1.7mL of the mixed reagents and values of ROS were recorded after an interval of 20sec using UV spectrophotometer at 505nm.

### Thiobarbituric acid reactive substance (TBARS)

TBARS was recorded as described earlier [85]. Briefly, the reaction mixture was prepared containing 100 μL each of Tris-HCl (150mM), ascorbic acid (1.5mM), ferrous sulphate (1.0mM) and tissue homogenate (100 μL) in 600 μL of distilled water. The mixture was vortexed and kept in incubator for 15 min at 37°C. Then, 1 mL trichloroacetic acid (10%) and thiobarbituric acid (0.375%) were added to the mixture, vortexed and then placed in the water bath (100°C) for 15 min. Finally, the contents were centrifuged at 1000g for 10 min,the supernatant was collected and the optical density was determined using UV visible spectrophotometer at 532nm.

### Reduced glutathione (GSH)

The quantity of GSH was recorded by following the earlier procedure [86]. Briefly, the reaction mixture containing100μL tissue homogenate, 1000 μL disodium hydrogen phosphate buffer and 500 μL DTNB reagent was prepared and optical density of the mixture was recorded using UV visible spectrophotometer at 412nm wavelength.

### Superoxide dismutase (SOD)

The concentration of SOD was measured by following the previous method [86,87]. For this purpose, 1500 μL L-methionine (9.9mM), 1000 μL nitroblue tetrazolium (NBT, 57.0μM) and 750 μL Triton X-100 (0.025%) were mixed in 30mL PBS (50mM,7.8 pH). Then, 90 μL of tissue homogenate was mixed in 1000 μL of prepared mixture. The mixture was then placed under fluorescent light for 10 min and then incubated at 37°C for 5 min. Finally, 10 μL of riboflavin (0.9μM) was added and the mixture was placed at 40°C for 10 min. Then, the values of SOD were measured by using UV visible spectrophotometer at 560nm.

### Catalase (CAT)

The quantity of CAT was recorded according to previous method [88]. Briefly, a reaction mixture having 100 μL tissue sample, 2mL potassium phosphate buffer (50mM, pH 7.0) and 1mL H_2_O_2_ (5.9mM) was prepared and the values of CAT were determined using UV visible spectrophotometer at 240nm.

### Peroxide (POD)

The quantity of POD was recorded using the method as reported earlier [88]. Briefly, a reaction mixture containing 75 μL of H_2_O_2_ (40mM), 25 μL of guaiacol (20mM), 625 μL of potassium phosphate buffer (50mM, pH 5.0) and 25 μL of tissue was prepared and the values of POD were measured by using UV spectrophotometer at 470nm.

### Statistical analysis

The obtained results were presented as mean±standard error of mean (SEM) of three independent observations in each experiment. Statistical analysis was performed using one-way analysis of variance (ANOVA) in IBM SPSS^®^ statistics software (version 20). The significant differences among the means were compared with Tukey’s post-hoc test at *p* < 0.05 level of significance.

## Results

### Clinical signs of toxicity

The animal health parameters and behavioral signs were monitored on daily basis and different clinical signs like loss of equilibrium, jerking movements, surface breathing and swimming on one side were observed in the fish exposed to higher concentrations of CuSO_4_ at days-24 and 36 of the experiment. However, since the doses of CuSO_4_selected for experimentation were quite low and of sub-lethal level, thus, no mortality was observed during the whole study period, even in the treated fish exposed to higher concentration (0.56 µg/L) of CuSO_4_ at the end of the experiment, i.e., at day-36.

### Morphological and nuclear changes in erythrocytes

**Fig 1:**
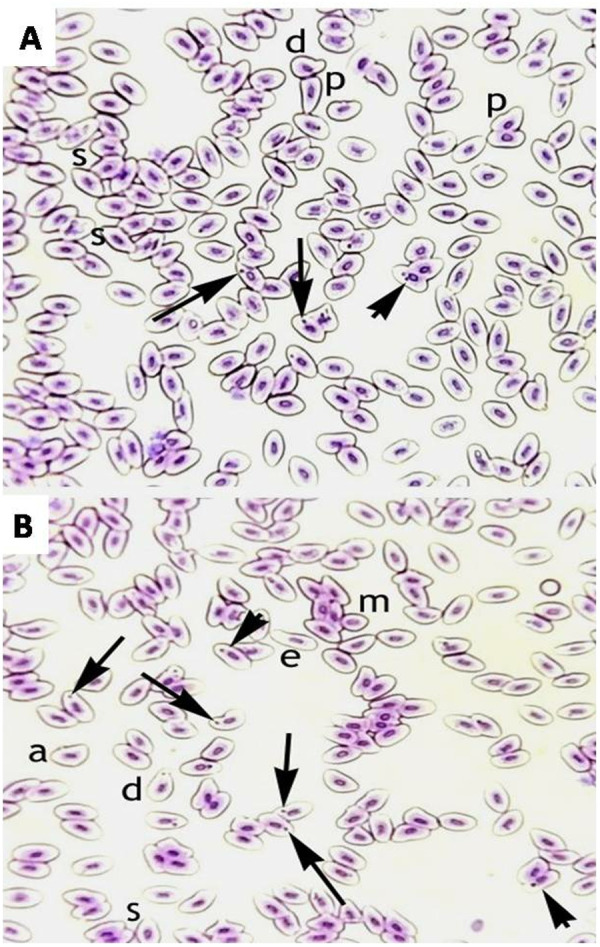
Photomicrograph of morphological abnormalities in the erythrocytes of freshwater fish (*Labeo rohita*). The fish were treated with CuSO_4_at 0.42 µg/L (A) and 0.56 µg/L (B) concentrations showing different aberrations in the erythrocytes like micronucleus (arrow), dividing cells (arrowhead), abnormal shaped cells (a), droplet cells (d), elliptocytes (e), spindle shaped cells (s), microcytes (m) and pear-shaped cells (p). Giemsa stain, 1000X (oil immersion lens).

**Fig 2:**
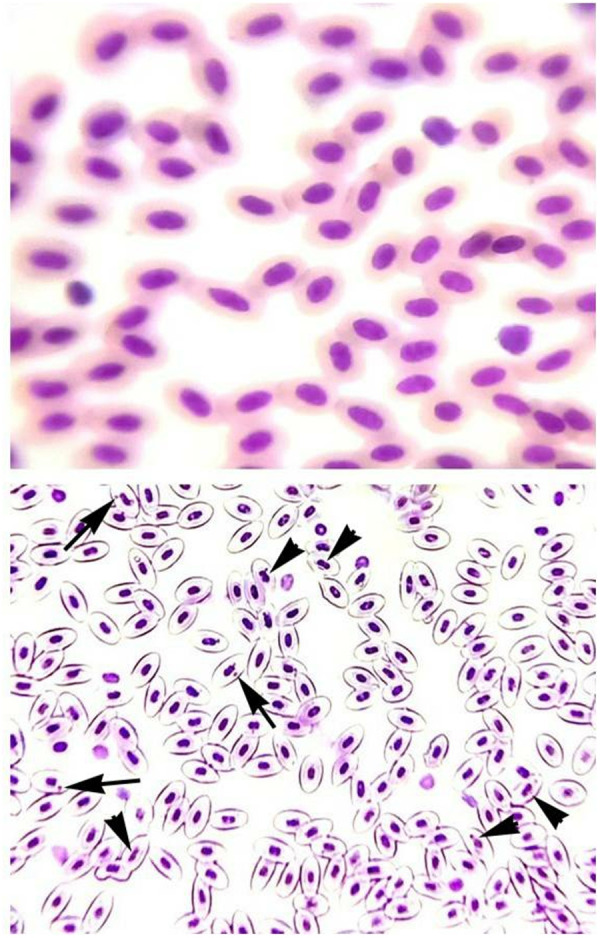
Photomicrograph of nuclear changes in the erythrocytes of *Labeo rohita.* The normal erythrocytes (A) of *Labeo rohita* became severely affected with different erythrocytic abnormalities (B) on treatment with CuSO_4_ (0.56 µg/L) and thus, showing nuclear changes micronucleus (arrow) and dividing nucleus (arrowhead). Giemsa stain, 1000X (oil immersion lens).

The frequency of different morphological and nuclear alterations in the erythrocytes subjected to different concentrations of CuSO_4_was found to be significantly (p < 0.05) higher in the fish exposed to 0.42 µg/L (group C) and 0.56 µg/L (group D) of CuSO_4_ (Table 2). A significantly (*p* < 0.05) increased number of micronuclei in the erythrocytes and abnormal shaped cells such as droplet cells, elliptocytes, spindle shaped cells, microcytes and pear-shaped cells were observed in the groups treated with 0.42 µg/L and 0.56 µg/L of CuSO_4_ (Fig 1). Moreover, in the fish treated with highest concentration of CuSO_4_ (0.56 µg/L), the erythrocytes exhibited abnormalities like dividing nucleus indicating regeneration along with the presence of micronucleus (Fig 2).

**Table 2:** Frequency of different morphological and nuclear changes in the erythrocytes of fish exposed to various concentrations of CuSO_4_.

Days	Groups			
	A (Control)	B (0.28 µg/L)	C (0.42 µg/L)	D (0.56 µg/L)
**Notched nucleus (%)**				
12	1.79 ± 0.01	1.85 ± 0.01	2.33 ± 0.08*	2.64 ± 0.10*
24	1.76 ± 0.01	1.89 ± 0.02	2.48 ± 0.09*	2.69 ± 0.07*
36	1.76 ± 0.01	1.84 ± 0.01	2.65 ± 0.11*	2.53 ± 0.09*
**Binucleate nucleus (%)**				
12	1.42 ± 0.17	1.48 ± 0.12	1.62 ± 0.08	1.71 ± 0.04
24	1.46 ± 0.19	1.57 ± 0.04	1.86 ± 0.09	3.08 ± 0.09*
36	1.47 ± 0.12	1.61 ± 0.06	3.88 ± 0.04*	3.09 ± 0.08*
**Micronucleus (%)**				
12	1.71 ± 0.17	1.81 ± 0.18	1.83 ± 0.07	1.91 ± 0.07
24	1.72 ± 0.23	1.94 ± 0.08	1.87 ± 0.12	4.02 ± 0.04*
36	1.73 ± 0.20	1.99 ± 0.12	3.72 ± 0.13*	4.23 ± 0.05*
**Condensed nucleus (%)**				
12	2.09 ± 0.56	2.22 ± 0.20	2.51 ± 0.65	2.39 ± 0.11
24	2.28 ± 0.32	2.33 ± 0.50	3.80 ± 0.15	4.16 ± 0.04*
36	2.43 ± 0.47	2.45 ± 0.12	4.93 ± 0.69*	6.25 ± 0.25*
**Spindle shaped erythrocyte (%)**				
12	1.42 ± 0.20	2.67 ± 0.14	3.20 ± 0.19*	4.37 ± 0.25*
24	1.48 ± 0.18	2.76 ± 0.18	3.32 ± 0.13*	4.96 ± 0.20*
36	1.52 ± 0.33	2.81 ± 0.32	3.47 ± 0.16*	5.01 ± 0.09*
**Spherocytes (%)**				
12	1.92 ± 0.02	2.46 ± 0.16	3.43 ± 0.07*	4.18 ± 0.22
24	1.96 ± 0.07	2.54 ± 0.14	3.52 ± 0.09*	4.42 ± 0.15
36	1.99 ± 0.02	2.69 ± 0.09	3.73 ± 0.15*	4.66 ± 0.30
**Erythrocytes with lobed nucleus (%)**				
12	1.38 ± 0.07	1.46 ± 0.05	1.76 ± 0.08	3.47 ± 0.17*
24	1.29 ± 0.01	1.41 ± 0.03	1.56 ± 0.07	3.23 ± 0.23*
36	1.30 ± 0.02	1.46 ± 0.03	1.74 ± 0.08	4.46 ± 0.17*
**Erythrocytes with blabbed nucleus (%)**				
12	1.21 ± 0.16	1.37 ± 0.21	1.52 ± 0.12	2.97 ± 0.11*
24	1.21 ± 0.14	1.46 ± 0.16	1.55 ± 0.10	3.16 ± 0.08*
36	1.18 ± 0.13	1.45 ± 0.15	2.60 ± 0.14*	3.16 ± 0.06*
**Erythrocytes with vacuolated nucleus (%)**				
12	2.34 ± 0.02	2.41 ± 0.01	2.61 ± 0.10	2.97 ± 0.05
24	2.35 ± 0.01	2.42 ± 0.03	2.63 ± 0.04	3.01 ± 0.09*
36	2.38 ± 0.02	2.45 ± 0.04	3.69 ± 0.05*	3.89 ± 0.15*
**Pear shaped erythrocyte (%)**				
12	3.65 ± 0.33	4.02 ± 0.17	5.58 ± 0.13*	7.05 ± 0.03*
24	3.77 ± 0.30	4.17 ± 0.15	6.66 ± 0.31*	7.11 ± 0.19*
36	3.64 ± 0.20	4.19 ± 0.42	6.73 ± 0.27*	7.15 ± 0.07*

The values (mean±SEM) are significantly (p < 0.05) increased in the fish exposed to 0.42 µg/L (group C) and 0.56 µg/L (group D) of CuSO4 compared to the control group. * = p < 0.05.

### Oxidative stress parameters and anti-oxidant enzymes

The oxidative stress profile (ROS, TBARS) and the concentrations of various anti-oxidant enzymes (GSH, SOD, CAT, POD) in the gills (Table 3), brain (Table 4), liver (Table 5) and kidneys (Table 6) tissues of fish were found to be significantly (p < 0.05) affected in the fish exposed to increased concentrations (0.42 µg/L; group C, 0.56 µg/L; group D) of CuSO_4_.The concentrations of ROS and TBARS were significantly (p < 0.05) increased in all these tissues in the groups C and D while GSH contents and concentrations of SOD, CAT and POD showed a significant (p < 0.05) reduction in all the organ tissues of these two treated groups(C and D) exposed to higher (0.42 µg/L, 0.56 µg/L) concentrations of CuSO_4_compared to the control group A.

**Table 3:** Oxidative stress parameters (ROS, TBARS) and anti-oxidant enzymes (GSH, SOD, CAT, POD) in the gills of *Labeo rohita* exposed to various concentrations of CuSO_4_.

Days	Groups/Treatments			
	A (Control)	B (0.28 µg/L)	C (0.42 µg/L)	D (0.56 µg/L)
**Reactive Oxygen Species (Optical density)**				
12	0.31 ± 0.02	0.32 ± 0.01	0.32 ± 0.03	0.52 ± 0.05*
24	0.33 ± 0.01	0.34 ± 0.03	0.48 ± 0.04*	0.55 ± 0.01*
36	0.32 ± 0.03	0.35 ± 0.01	0.50 ± 0.05*	0.57 ± 0.05*
**Thiobarbituric Acid Reactive Substances (nmol TBARS/h/mg protein)**				
12	40.65 ± 1.42	41.45 ± 3.62	42.25 ± 2.62	52.05 ± 2.12*
24	41.11 ± 1.56	42.86 ± 2.65	47.62 ± 2.61*	55.37 ± 1.21*
36	41.18 ± 2.62	43.02 ± 1.62	48.80 ± 1.62*	57.68 ± 0.62*
**Reduced glutathione (μmol GSH/mg protein)**				
12	2.56 ± 0.03	2.49 ± 0.03	2.44 ± 0.06	2.39 ± 0.03
24	2.42 ± 0.02	2.37 ± 0.06	2.35 ± 0.05	1.99 ± 0.02*
36	2.45 ± 0.04	2.34 ± 0.08	1.74 ± 0.09*	1.95 ± 0.12*
**Superoxide dismutase (units/mg protein)**				
12	10.77 ± 0.28	9.71 ± 0.12	9.66 ± 0.21	9.63 ± 0.31
24	10.66 ± 0.24	9.51 ± 0.29	9.35 ± 0.23	7.20 ± 0.19*
36	10.55 ± 0.32	9.30 ± 0.23	9.05 ± 0.25	6.80 ± 0.11*
**Catalase (units/mg protein)**				
12	3.01 ± 0.14	2.98 ± 0.15	2.86 ± 0.07	2.19 ± 0.04*
24	2.96 ± 0.09	2.88 ± 0.12	2.71 ± 0.05	2.13 ± 0.08*
36	2.93 ± 0.08	2.85 ± 0.10	2.37 ± 0.03*	2.09 ± 0.06*
**Peroxidase (units/mg protein)**				
12	0.39 ± 0.07	0.35 ± 0.04	0.34 ± 0.06	0.34 ± 0.09
24	0.38 ± 0.03	0.33 ± 0.06	0.33 ± 0.04	0.24 ± 0.08*
36	0.37 ± 0.06	0.32 ± 0.11	0.27 ± 0.02*	0.22 ± 0.05*

The values (Mean±SEM) of ROS and TBARS were significantly (p < 0.05) increased while a significant (p < 0.05) decrease in the values of anti-oxidative enzymes in the fish exposed to higher concentrations (group C: 0.42 µg/L and group D: 0.56 µg/L) of CuSO4 compared to the control group A. * = p < 0.05.

**Table 4:** Oxidative stress parameters (ROS, TBARS) and anti-oxidant enzymes (GSH, SOD, CAT, POD) in the brain of *Labeo rohita* exposed to various concentrations of CuSO_4_.

Days	Treatments			
	A (Control)	B (0.28 µg/L)	C (0.42 µg/L)	D (0.56 µg/L)
**Reactive Oxygen Species (Optical density)**				
12	0.39 ± 0.01	0.41 ± 0.02	0.42 ± 0.02	0.44 ± 0.02
24	0.40 ± 0.03	0.43 ± 0.01	0.62 ± 0.09*	0.71 ± 0.03*
36	0.41 ± 0.02	0.44 ± 0.03	0.66 ± 0.06*	0.73 ± 0.09*
**Thiobarbituric Acid Reactive Substances (nmol TBARS/h/mg protein)**				
12	18.58 ± 2.16	19.27 ± 1.26	21.15 ± 2.28	21.64 ± 3.60
24	19.06 ± 2.18	21.75 ± 2.13	26.43 ± 3.17*	30.12 ± 1.33*
36	19.16 ± 3.21	22.90 ± 1.11	26.64 ± 2.36*	30.38 ± 2.36*
**Reduced glutathione (μmol GSH/mg protein)**				
12	2.98 ± 0.11	2.88 ± 0.01	2.77 ± 0.07	2.71 ± 0.07
24	2.93 ± 0.15	2.74 ± 0.03	2.14 ± 0.05*	1.75 ± 0.11*
36	2.86 ± 0.13	2.68 ± 0.01	2.09 ± 0.08*	1.71 ± 0.10*
**Superoxide dismutase (units/mg protein)**				
12	13.63 ± 2.11	12.97 ± 2.02	11.95 ± 1.09	8.94 ± 0.33*
24	13.54 ± 1.13	12.68 ± 1.15	10.21 ± 1.31*	8.85 ± 1.23*
36	13.43 ± 2.12	12.46 ± 1.23	10.03 ± 0.28*	8.73 ± 1.25*
**Catalase (units/mg protein)**				
12	4.14 ± 0.18	3.94 ± 0.28	3.89 ± 0.18	3.86 ± 0.13
24	4.10 ± 0.29	3.86 ± 0.07	3.78 ± 0.11	2.48 ± 0.05*
36	4.05 ± 0.21	3.85 ± 0.15	2.36 ± 0.21*	2.24 ± 0.09*
**Peroxidase (units/mg protein)**				
12	3.10 ± 0.22	2.97 ± 0.11	2.92 ± 0.05	2.87 ± 0.06
24	3.09 ± 0.16	2.91 ± 0.07	2.83 ± 0.03	1.96 ± 0.36*
36	3.02 ± 0.13	2.90 ± 0.05	2.29 ± 0.06*	1.92 ± 0.27*

The values (Mean±SEM) of oxidative stress parameters were significantly (p < 0.05) increased while a significant (p < 0.05) decrease in the values of anti-oxidative enzymes was observed in the fish of the groups C (0.42 µg/L) and D (0.56 µg/L) compared to the control group. * = p* < *0.05.

**Table 5:** Oxidative stress parameters (ROS, TBARS) and levels of anti-oxidant enzymes (GSH, SOD, CAT, POD) in the liver of *Labeo rohita* exposed to various concentrations of CuSO_4_.

Days	Treatments			
	A (Control)	B (0.28 µg/L)	C (0.42 µg/L)	D (0.56 µg/L)
**Reactive Oxygen Species (Optical density)**				
12	0.34 ± 0.03	0.35 ± 0.02	0.38 ± 0.03	0.85 ± 0.07*
24	0.35 ± 0.06	0.37 ± 0.04	0.69 ± 0.11*	0.86 ± 0.04*
36	0.38 ± 0.04	0.41 ± 0.03	0.73 ± 0.02*	0.91 ± 0.05*
**Thiobarbituric Acid Reactive Substances (nmol TBARS/h/mg protein)**				
12	37.39 ± 2.94	39.23 ± 1.94	41.07 ± 1.14	54.91 ± 3.74*
24	38.87 ± 1.93	40.66 ± 2.93	50.44 ± 1.99*	56.23 ± 2.91
36	40.02 ± 3.96	41.95 ± 2.95	51.88 ± 2.35*	58.80 ± 4.55*
**Reduced glutathione (μmol GSH/mg protein)**				
12	8.64 ± 1.16	7.63 ± 0.56	6.62 ± 0.16	5.61 ± 0.16*
24	8.36 ± 1.11	7.42 ± 1.10	6.01 ± 0.61*	5.54 ± 0.11*
36	8.31 ± 1.13	7.36 ± 1.05	5.91 ± 1.15*	5.46 ± 0.15*
**Superoxide dismutase (units/mg protein)**				
12	11.63 ± 0.33	10.95 ± 1.11	10.27 ± 0.81	10.25 ± 0.56
24	11.68 ± 0.14	10.31 ± 0.74	9.44 ± 0.14	7.56 ± 0.64*
36	10.72 ± 0.17	9.65 ± 1.17	7.08 ± 0.19*	7.02 ± 0.17*
**Catalase (units/mg protein)**				
12	8.68 ± 0.26	7.50 ± 0.15	7.12 ± 0.28	5.15 ± 0.19*
24	7.99 ± 1.16	7.33 ± 0.52	5.61 ± 0.46*	5.02 ± 0.32*
36	7.93 ± 0.86	6.99 ± 0.76	5.44 ± 0.32*	4.94 ± 0.71*
**Peroxidase (units/mg protein)**				
12	4.06 ± 1.09	3.52 ± 0.51	2.78 ± 0.19*	2.45 ± 0.09*
24	4.01 ± 0.38	3.47 ± 0.29	2.61 ± 0.11*	2.41 ± 0.15*
36	3.94 ± 0.59	3.39 ± 0.32	2.53 ± 0.22*	2.29 ± 0.16*

The values (Mean±SEM) of ROS and TBARS were significantly (p < 0.05) increased while a significant (p < 0.05) decrease in the level of anti-oxidative enzymes was found in the fish of the groups C (0.42 µg/L) and D (0.56 µg/L) compared to the control group A. * = p < 0.05.

**Table 6:** Oxidative stress parameters (ROS, TBARS) and concentrations of anti-oxidant enzymes (GSH, SOD, CAT, POD) in the kidneys of *Labeo rohita* exposed to various concentrations of CuSO_4_.

Days	Treatments			
	A (Control)	B (0.28 µg/L)	C (0.42 µg/L)	D (0.56 µg/L)
**Reactive Oxygen Species (Optical density)**				
12	0.48 ± 0.11	0.51 ± 0.24	0.53 ± 0.11	0.70 ± 0.16*
24	0.51 ± 0.16	0.54 ± 0.16	0.67 ± 0.20*	0.75 ± 0.28*
36	0.54 ± 0.13	0.58 ± 0.21	0.70 ± 0.13*	0.78 ± 0.21*
**Thiobarbituric Acid Reactive Substances (nmol TBARS/h/mg protein)**				
12	28.49 ± 1.56	32.19 ± 1.26	32.90 ± 1.19	39.61 ± 2.53*
24	29.06 ± 1.62	33.05 ± 1.38	37.05 ± 1.61*	41.04 ± 2.26*
36	29.65 ± 1.71	33.71 ± 1.80	39.77 ± 1.34*	41.83 ± 2.71*
**Reduced glutathione (μmol GSH/mg protein)**				
12	7.68 ± 1.01	6.43 ± 0.59	6.18 ± 0.18	4.93 ± 0.29*
24	7.60 ± 1.16	6.36 ± 1.41	5.11 ± 0.26*	4.87 ± 0.22*
36	7.53 ± 1.32	6.30 ± 1.02	5.07 ± 0.38*	4.35 ± 0.31*
**Superoxide dismutase (units/mg protein)**				
12	15.49 ± 1.32	13.91 ± 1.21	13.83 ± 1.12	9.55 ± 0.32*
24	15.28 ± 1.43	13.10 ± 1.05	10.93 ± 0.95*	8.75 ± 0.65*
36	15.26 ± 1.36	13.07 ± 1.13	10.87 ± 1.01*	8.68 ± 0.46*
**Catalase (units/mg protein)**				
12	5.10 ± 1.09	4.56 ± 0.91	4.42 ± 0.49	3.48 ± 0.22*
24	5.07 ± 1.11	4.53 ± 0.72	3.99 ± 0.57*	3.45 ± 0.19*
36	5.02 ± 1.07	4.46 ± 0.29	3.91 ± 0.63*	3.35 ± 0.27*
**Peroxidase (units/mg protein)**				
12	5.96 ± 0.31	5.31 ± 0.12	4.95 ± 0.13	4.01 ± 0.11*
24	5.92 ± 0.21	5.26 ± 0.27	4.61 ± 0.16*	3.95 ± 0.21*
36	5.87 ± 0.13	5.22 ± 0.05	4.56 ± 0.19*	3.91 ± 0.21*

The values (Mean±SEM) of oxidative stress parameters were significantly (p < 0.05) increased while the level of anti-oxidative enzymes was significant (p < 0.05) decreased in the fish exposed to higher concentrations (group C: 0.42 µg/L, group D: 0.56 µg/L) of CuSO4 compared to the control group. * = p < 0.05.

### DNA damage by Comet assay

**Fig 3:**
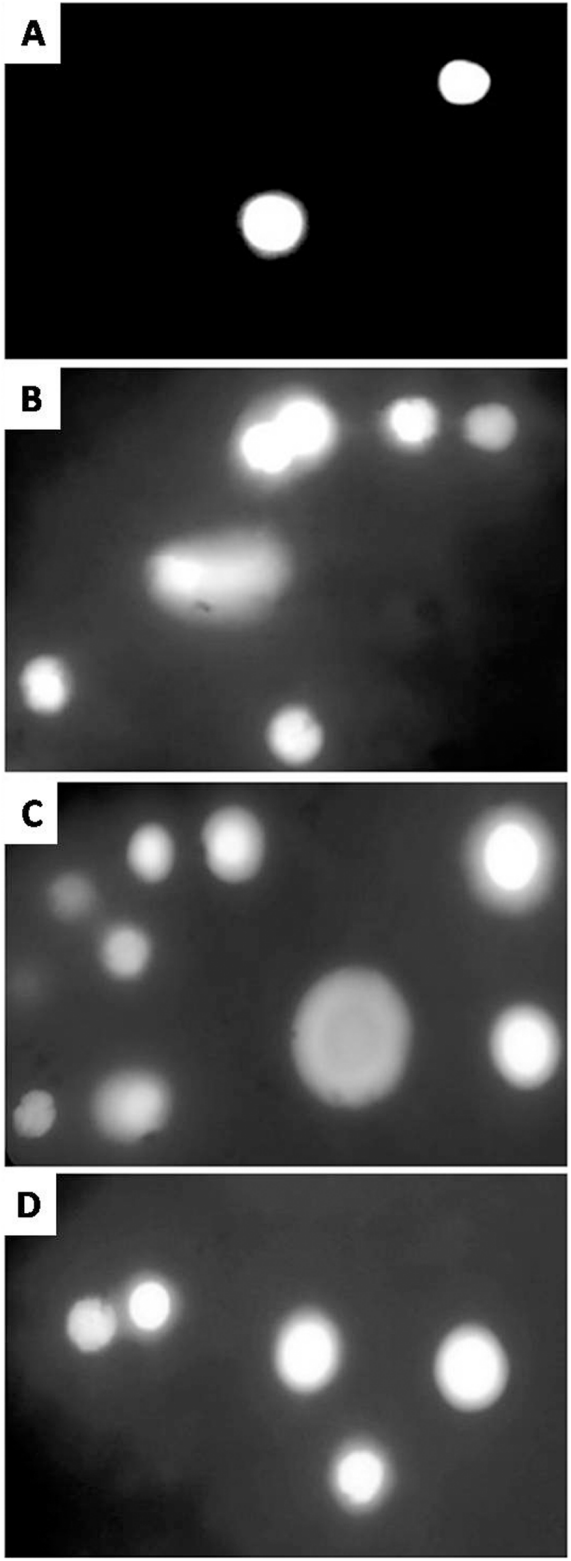
Photomicrograph of comet assay. The normal cells (A) compared to DNA damage in the treated cells: hepatocytes (B), isolated kidney cells (C) and brain cells (D) of fish exposed to higher concentration (0.56 µg/L) of CuSO4. The normal cells are depicting the intact DNA while the treated cells illustrate the DNA damage. The intensity of fluorescence around the nucleus depends upon the degree of damage and increase with an increase in the concentration of the treatment. Ethidium bromide staining; 400X.

**Fig 4:**
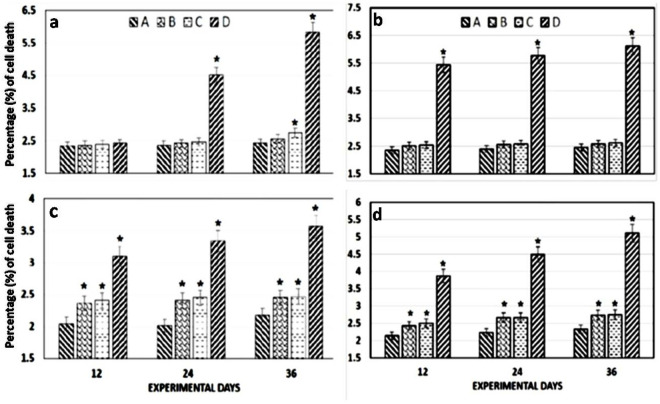
Comet assay (% of cell damage) in the leukocytes. The cells of (a), brain (b), liver (c) and kidneys (d) of the control (Group A) and CuSO_4_ treated groups (Group B: 0.28 µg/L; Group C: 0.42 µg/L; Group D: 0.56 µg/L). The severity of DNA damage has found to be significantly (p < 0.05) increased with an increase in the dose of CuSO_4_ and the duration of exposure. Bars are showing mean±SEM values. The bars bearing asterisk differ significantly than the control group A, * = p < 0.05.

A significant damage to the DNA of lymphocytes (Fig 3A), hepatocytes (Fig 3B), isolated kidney cells (Fig 3C) and brain cells (Fig 3D) of fish was observed at highest (0.56 µg/L) treatment concentration of CuSO_4_. The intensity of fluorescence around the nucleus depicts the severity of the damage and thus, an increase in the fluorescence around the nuclei has been observed with an increase in the concentration of the treatment (Fig 3). The severity of damage in the DNA of leukocytes (Fig 4a), brain (Fig 4b), liver (Fig 4c) and kidneys (Fig 4d) of *Labeo rohita* increased with an increase in the dose of CuSO_4_ and the duration of exposure (Fig 4). A significantly (*p* < 0.05) higher DNA damage was occurred in all the tissues of fish of the group D (0.56 µg/L) as compared to the control group A. It has been observed that with an increase in the concentration of CuSO_4_, the damage in these tissues was increased with the passage of time and thus, the greatest damage was recorded at the highest dose, at day-36 of the trial (Fig 4).

### Histo-pathological observations

**Fig 5:**
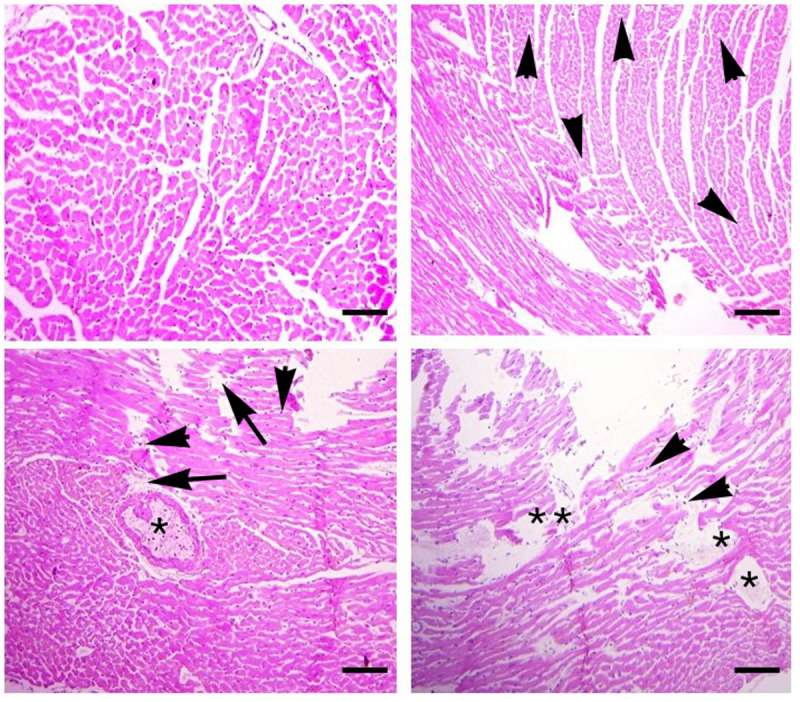
Photomicrograph of different sections of heart of *Labeo rohita.* The fish were exposed to different concentrations of CuSO_4_ at days-36 of the experiment. A) normal histological arrangements of cardiac tissue; B) vacuolar degeneration (arrow heads); C) edema (*), necrosis of cardiac myocytes (arrow heads) and myofibrillosis (arrow); D) edema (*), necrosis of cardiac myocytes (arrow heads) and myofibrillosis (**). Scale bar: 50μm. Hematoxylin and Eosin Staining. Left panels: 400X, Right panels: 200X.

The histo-pathological alterations in the heart of fish exposed to higher levels of CuSO_4_ were neutrophilic infiltration, vacuolar degeneration, necrosis of cardiac myocytes, myofibrillosis, edema, inflammation, hemorrhages and deposition of fibrin were observed in the fish exposed to higher concentrations of CuSO_4_(0.42 µg/L and 0.56 μg/L) at days-24 and 36 of the experiment (Fig 5). The lesions were observed to be mild in the fish treated with lower dose of CuSO_4_ (0.28 µg/L) and at the start of the experiment (days-12), however the abnormalities were found to be significantly increased with an increase in the concentration of the treatment and duration of the experiment, i.e., days-24 and 36 (Table 7).

**Table 7:** Severity of various histo-architectural alterations in the heart of *Labeo rohita* exposed to different doses of copper sulphate.

Histo-pathological lesions/days	Treatments			
	A (Control)	B (0.28 µg/L)	C (0.42 µg/L)	D (0.56g/L)
**Day 12**				
Necrosis of cardiac myocytes	–	+	+	+
Vacuolar Degeneration		+	+	++
Myofibrillosis	–	+	+	++
Neutrophilic myocarditis	–	+	++	++
Edema	–	+	++	++
Hemorrhages	–	–	+	+
Disorganization of cardiac myofibers	–	+	++	++
Eccentric nuclei of cardiac myocyte	–	+	+	++
**Day 24**				
Necrosis of cardiac myocytes	–	+	++	+++
Vacuolar Degeneration		+	++	++++
Myofibrillosis	–	+	+	+++
Neutrophilic myocarditis	–	+	++	+++
Edema	–	+	++	+++
Hemorrhages	–	–	++	++
Disorganization of cardiac myofibers	–	+	+++	++++
Eccentric nuclei of cardiac myocyte	–	+	+++	++++
**Day 36**				
Necrosis of cardiac myocytes	–	+	+++	++++
Vacuolar Degeneration		++	++++	++++
Myofibrillosis	–	+	+++	++++
Neutrophilic myocarditis	–	++	+++	++++
Edema	–	+	+++	++++
Hemorrhages	–	–	++	+++
Disorganization of cardiac myofibers	–	+	+++	++++
Eccentric nuclei of cardiac myocyte	–	+	+++	++++

The control group A did not show any pathological lesions. Mild: + = 25%, moderate: ++ = 50%, severe:+++ = 75%, much severe:++++ = 100%.

## Discussion

Copper has been found to cause patho-physiological disorders, oxidative stress, inflammation and toxicity in *Labeo rohita* [55,65,89] and *Cyprinus carpio* [90]. Copper poisoning results in significant increase in WBCs, blood glucose and cholesterol and levels of serum enzymes, i.e., alanine aminotransferase, aspartate aminotransferase, alkaline phosphatase, lactate dehydrogenase, while erythrocyte count, Hb concentration, hematocritand serum total proteins, albumin and globulin were significantly decreased that clearly indicatea decline in the health of freshwater fish *Labeo rohita* [65].In a previous study, the genotoxic effects of water of the Helong Reservoir (China) were studied on four common fish species (*Labeo rohita*, *Cirrhinus molitorella*, *red tilapia* and *Oreochromis niloticus*) present in the reservoir, wherein, no significant difference were observed in different parameters (DNA damage) among these four species as compared to the control [33]. It depicts that the toxicity effects are comparable among these four species, hence, we selected *Labeo rohita*, as a representative fish species in the current study that is also a commonly available fish in our region.

In our study, erythrocytes with notched nucleus, binucleated, condensed, spindle shaped and spherocytes were observed in *Labeo rohita* exposed to CuSO_4_ and the damage was found to be increased with as increase in the concentration of the treatment and the time of exposure. These significantly higher frequency of nuclear abnormalities like lobbed, blebbed, vacuolated and pear-shaped nuclei found in the erythrocytes are most likely due to toxicity stress caused by CuSO_4_ leading to severe alterations in the nuclear and cellular morphology. Formerly, it was observed that metal pollutants like Cu caused a significant increase in the frequency of micronuclei in *Channa punctatus* [57] and *Pagrus major* [91]. Similarly, different nuclear aberrations have been observed in the fish erythrocytes including nuclear remnants, micronuclei, anucleated, notched, condensed and blebbed nuclei as well asmorphological alterations like leptocytes, vacuolated nucleus, stomatocytes and tear shaped cells have also been observed subjected to thiamethoxam [92], fipronil [93] and cadmium [94]. Similarly, likewise that of copper, other metals also found to cause nuclear alterations in the blood cells and histo-morphological modifications in different organs of *Ctenopharyngodon idella* [95] and *Labeo rohita* [96].Similarly, it was observed that CuSO_4_ decreased the erythrocytes count, Hb values and hematocrit in *Oreochromis niloticus* [97]. The binucleated and pear-shaped erythrocytes, notched nuclei, lobed, blebbed nuclei and micronuclei have also been found in the erythrocytes of grass carp [36] zebrafish [98] common carp *Cyprinus carpio* [99], Wistar rats [100] and other avian and mammalian species [101] on treatment with insecticides (pyrethroid) that might cause mitochondrial oxidative damage leading to oxidative stress.

The level of oxidative stress parameters like ROS and TBARS in the gills, liver, brain and kidneys of fish subjected to CuSO_4_ were significantly elevated in a time- and dose-dependent manner in our study. Under the treatment of different doses of CuSO_4_, initially there observed a non-significant increase in the level of ROS and TBARS at low dose (0.28 µg/L) of CuSO_4_, the level further increased at high concentration (0.42 µg/L) and became significantly (p < 0.05) adverse at higher concentration (0.56 µg/L) of CuSO_4_. The same is true for decrease in the concentrations of SOD, POD, GSH and CAT, wherein the decrease was mild at lower doses (0.28 µg/L, 0.42 µg/L) of CuSO_4_and at the start or mid of the experiment (day-24) and then their levels were significantly (p < 0.05) decreased at higher (0.56 µg/L) dose of CuSO_4_at the end of the experiment, i.e., day-36. Furthermore, owing to the detoxifying systems of exposed animals, exposure of diverse species to various toxicants produces faster and greater production of ROS from various visceral organs of fish like liver, gills and muscles [102] and thus, the production of ROS stimulates the denaturation of proteins and lipid peroxidation system that lead to cell membrane defects and thus, the production of TBARS [103]. Various contaminants and toxicants have also been reported to elevate the level of oxidative stress indices such as product of lipid peroxidation, ROS and nitric oxide in juvenile red sea bream [91] and rats [104,105]. Likewise, CuSO_4_ was found to decrease SOD and CAT activities while elevated the glutathione and malondialdehyde levels in *Oreochromis niloticus* [106]. The total proteins and GSH contents in the gills, liver, brain and kidney tissues of treated fish have been found to be lower in the current investigation that might be attributed to altered tissue functioning, since the cadmium toxicity also leads to oxidative stress in *Labeo rohita* and thus, a decrease in GSH contents in the hepatic tissue [107]. Moreover, the same has also been previously reported that the oxidative damage was caused in the liver tissue after being exposed to heavy metals (Cd, Cu, Cr, Pb, Zn) in *Labeo rohita* [108,109]. Similarly, lower levels of GSH have been observed in the hepatic tissue of Delta Smelt [110] and Jundiara [111] fish and in the plasma of goldfish [112]. In the same context, the reduced amounts of these anti-oxidant enzymes in the gills, liver, brain and kidneys seen in this study might be related to CuSO_4_-induced generation of free radicals in these organs, as previously elaborated [113].

The excessive synthesis of free radicals like ROS causes oxidative damage that activates the body defense system to counteract the toxic impact of these radicals including stimulation of glutathione reductase, glutathione-S-transferase, SOD, GPx and CAT enzymes, as well as other compounds associated with anti-oxidant defense mechanism [114–116]. It is also documented that CuSO_4_ and CuO-NPs alone or in combination decrease the plasma total proteins and tissue SOD, CAT, glutathione-S-transferase, glutathione reductase and glutathione in *Oreochromis niloticus* [106]and zebrafish [117]. In the same context, in the current investigation, the toxicity stress of CuSO_4_considerably decreased the concentration of anti-oxidant enzymes (POD, SOD, CAT) in visceral organs of the fish in a dose-dependent manner as likely the reduction was observed in the concentrations of CAT, POD and SOD in the hepatic tissue of *Oreochromis niloticus*, when exposed to CuSO_4_ [118]and hence, is coherent with the results of our study.

The comet test is usually used to assess the metal-induced substantial changes in the level of DNA damage and the method successfully screens a wide range of genetic pollutants in the blood of *Oreochromis niloticus* [119], *Cyprinus carpio* [120], *Labeo rohita* [56], *Aristichthys nobilis* [121]and four fresh water fish; *Labeo rohita*, *Cirrhinus molitorella*, *red tilapia* and *Oreochromis niloticus* [33]. In the same context, it has been observed that CuSO_4_ caused an increase in DNA damage in *Labeo rohita* in a dose-dependent manner in the current study (Fig 4) that has also previously been reported that CuSO_4_ has genotoxic capacity to interact with DNA and causes detrimental changes in the mammalian cells as measured by an increase in the comet percentage [122]. The DNA damage is happened in a time and concentration related manner, with the most damage occurred at the lethal concentration [123,124] and the greatest DNA damage was seen at the highest concentration of the treatment [125,126]; thus, these observations equally support our findings.

The results of the current study indicate that CuSO_4_at sub-lethal concentrations causes a significant increase in the oxidative stress profile in different organs (gills, liver, brain and kidneys) and significant decrease in the anti-oxidant enzymes along with DNA damage in different cells in a time- and dose-dependent manner. Previously, as a part of the same research project, we observed that CuSO_4_ significantly decreased the blood cells (RBCs count, packed cells volume) and Hb concentration [65] that might be due to DNA damage and morphological alterations in the erythrocytes of the treated fish, as we have observed in the current study. Similarly, the previously found histo-pathological alterations in the gills, liver and kidneys of the treated fish [65] has now been confirmed by imbalance in the concentrations of stress parameters and the level of anti-oxidant enzymes in these organs. These toxic effects are also further depicted by neutrophilic infiltration at the inflammatory site, degeneration of the affected tissue and necrosis of the cells, as we have observed for cardiac myocytes in the histo-pathological observations. The effects of CuSO_4_ on different studied parameters were found to be mild to moderate at low (0.28 µg/L) to high (0.42 µg/L) concentrations of CuSO_4_ at the start (day-12) or mid (day-24) of the experiment, while these effects become adverse at higher (0.56 µg/L) concentration up-to the end of the experiment, i.e., day-36. The decreased number of erythrocytes, severe morphological alterations along with a decrease in the oxygen carrying capacity (level of hemoglobin) of these blood cells leading to severe health effects and thus greatly affects the growth performance of the fish. That is ultimately depicted by an overall decrease in the body weight of the treated fish on increasing the dose of CuSO_4_ and duration of the experiment and hence, clearly depicts the toxic effects of CuSO_4_ on the health status and thus, eventually affects the production potential of the fish.

## Conclusions

The results indicated that copper sulphate induces significant tissue damage in freshwater fish (*Labeo rohita*) by causing severe morphological and nuclear changes in the erythrocytes and a significant increase the level of oxidative stress parameters (ROS, TBARS),while a significant decrease in the anti-oxidant enzymes (SOD, GSH,POD,CAT) in these organs. These hemato-biochemical parameters are imperative biomarkers used to analyze the physiological condition of the fish and to assess the degree of damage to its vital organs. Hence, it could be concluded that CuSO_4_ is a hazardous compound that causes severe physio-biochemical disruptions in different organ systems of the fish by entering in to the food chain, even at quite low environment relevant and sub-lethal concentrations and thus, could ultimately affects the human health on consumption of these affected fish. It is therefore recommended to avoid the discharge of extensively used pesticides and waste materials of different industries in the canal or river waters that thus becomes severely harmful for natural inhabitants of freshwater environment.

## Supporting information

S1 File CuSO_4_ data tables. (XLS)
